# Authors' Reply to PDI‐2024‐0087: Letter to the Editor for “Metronomic Chemotherapy in Pediatric Neuroblastoma”

**DOI:** 10.1002/pdi3.70008

**Published:** 2025-05-21

**Authors:** Chao Yang

**Affiliations:** ^1^ Department of Surgical Oncology National Clinical Research Center for Child Health and Disorders Ministry of Education Key Laboratory of Child Development and Disorders China International Science and Technology Cooperation Base of Child Development and Critical Disorders Children's Hospital of Chongqing Medical University Chongqing China

Dear Editor:

We would like to thank Ivanovski P et al. for their positive comments on our recent publication [1]. Meanwhile, we are pleased to see that the authors are able to identify this early piece of work [2], which would be overlooked otherwise due to its lack of PubMed indexing.

## Conflicts of Interest

The author declares no conflicts of interest.

## Data Availability

Data sharing is not applicable to this article, as no new data were created or analyzed in this study.
